# Supplementary material for: Comparison of Two Schizophyllum commune Strains in Production of Acetylcholinesterase Inhibitors and Antioxidants from Submerged Cultivation
Source: J Fungi (Basel). 2021 Feb 4;7(2):115. doi: 10.3390/jof7020115 (PMC7913866; doi:10.3390/jof7020115)
Supplement: Supplementary file 1 [file jof-07-00115-s001.pdf]

Figure S1. Growth curve of mycelia and lyophilized filtrate for *S. commune* isolate from Italy (IT)

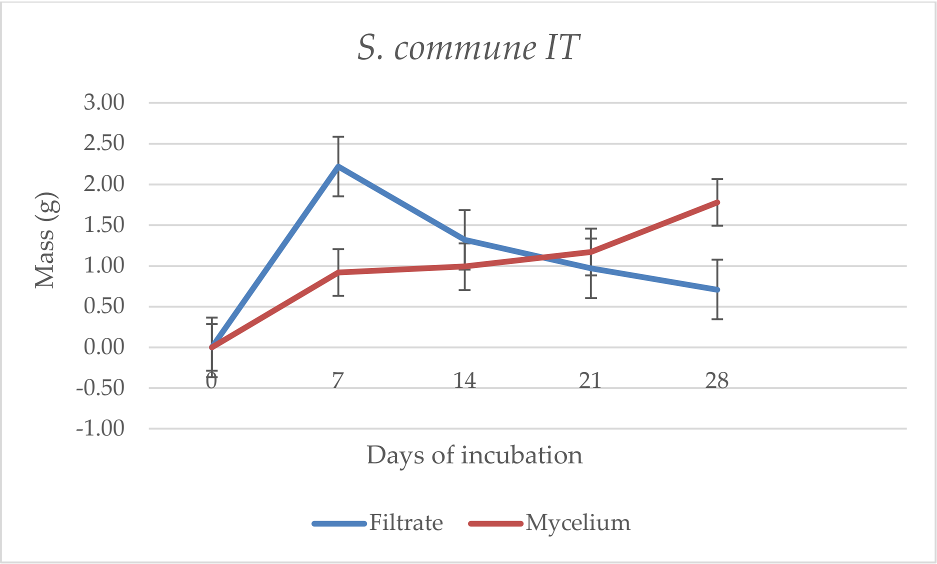

Figure S2. Growth curve of mycelia and lyophilized filtrate for *S. commune* isolate from Serbia (SRB)

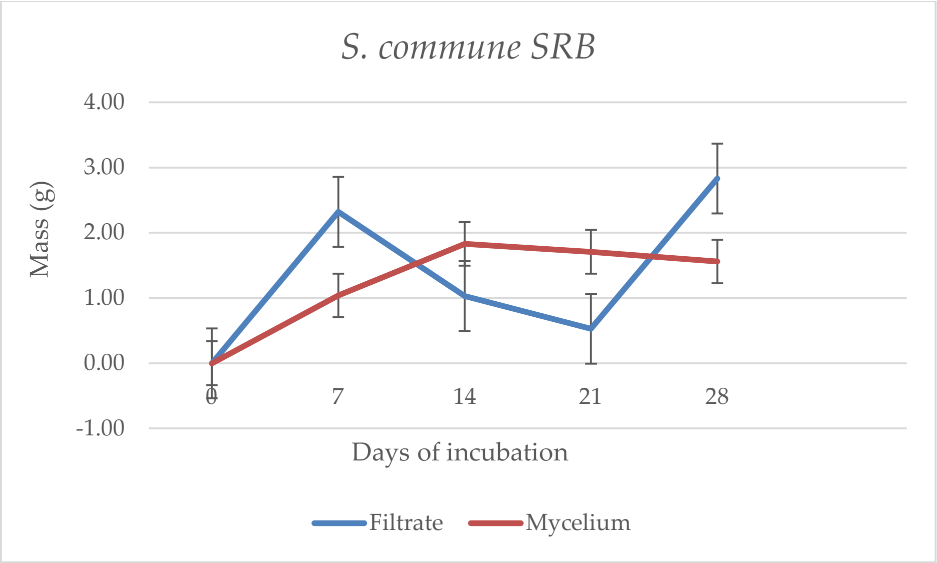

Figure S3. Fourier-transform infrared spectroscopy of ethanol samples of *S. commune* isolates from Serbia (SRB) and Italy (IT). Type of extracts examined: A′ – filtrate, 28 days, SRB; B′ – mycelium, 14 days, SRB; C′ – filtrate, 14 days, IT; D′ – mycelium, 14 days, IT.

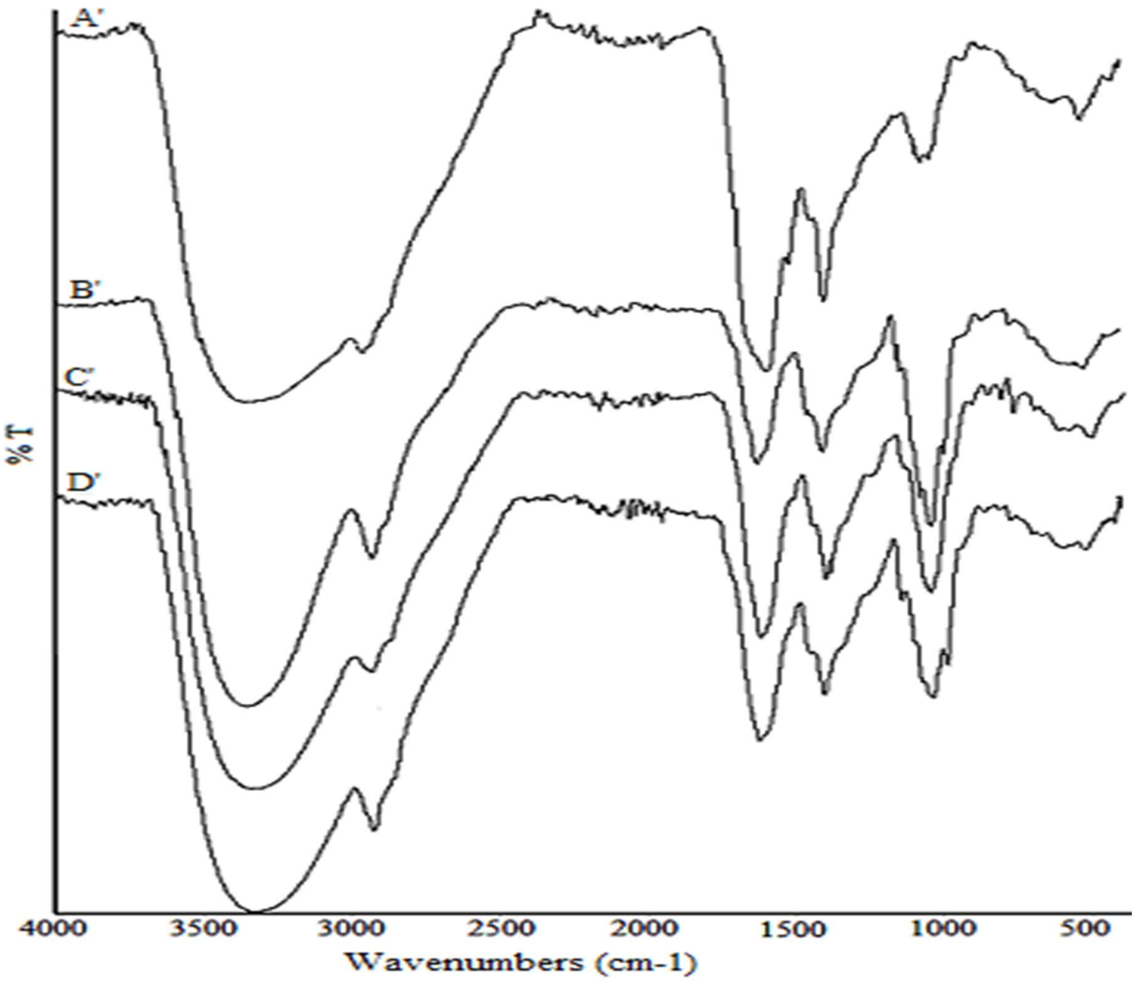

Table S1. Determined concentrations of phenolic compounds using LC-MS/MS technique in examined polysaccharide and ethanolic extracts (ng/mL). Concentrations are lower than the LoQ (limit of quantification), but higher than the LoD (limit of detection).

Scopoletin	Caffeic acid	Esculetin	Vanillic acid	o-coumaric acid	p-coumaric acid	Umbeliferon	2,5-dihydroxy benzoic acid	Cinnamic acid
<3.05	<6.1	<6.1	<48.85	<3.05	<3.05	<3.05	<3.05	<97.5
<3.05	<6.1	<6.1	<48.85	<3.05	<3.05	<3.05	<3.05	<97.5
<3.05	<6.1	<6.1	<48.85	<3.05	<3.05	<3.05	<3.05	<97.5
<3.05	<6.1	<6.1	<48.85	<3.05	<3.05	<3.05	<3.05	<97.5
<3.05	<6.1	<6.1	<48.85	<3.05	<3.05	<3.05	<3.05	<97.5
<3.05	<6.1	<6.1	<48.85	<3.05	<3.05	<3.05	<3.05	<97.5
<3.05	<6.1	<6.1	<48.85	<3.05	<3.05	<3.05	<3.05	<97.5
<3.05	<6.1	<6.1	<48.85	<3.05	<3.05	<3.05	<3.05	<97.5
<3.05	<6.1	<6.1	<48.85	<3.05	<3.05	<3.05	<3.05	<97.5
<3.05	<6.1	<6.1	<48.85	<3.05	<3.05	<3.05	<3.05	<97.5
<3.05	<6.1	<6.1	<48.85	<3.05	<3.05	<3.05	<3.05	<97.5
<3.05	<6.1	<6.1	<48.85	<3.05	<3.05	<3.05	<3.05	<97.5
<3.05	<6.1	<6.1	<48.85	<3.05	<3.05	<3.05	<3.05	<97.5
<3.05	<6.1	<6.1	<48.85	<3.05	<3.05	<3.05	<3.05	<97.5
<3.05	<6.1	<6.1	<48.85	<3.05	<3.05	<3.05	<3.05	<97.5
<3.05	<6.1	<6.1	<48.85	<3.05	<3.05	<3.05	<3.05	<97.5

Kaempferol	Luteolin	Naringenin	Bajkalein	Apigenin	Genistein	Daidzein	Synaptic acid	3,4-dimethoxy cinnamic acid	Syringic acid	Ferulic acid
<12.2	<24.4	<3.05	<195.5	<6.1	<3.05	<24.4	<48.85	<24.4	<12.2	<12.2
<12.2	<24.4	<3.05	<195.5	<6.1	<3.05	<24.4	<48.85	<24.4	<12.2	<12.2
<12.2	<24.4	<3.05	<195.5	<6.1	<3.05	<24.4	<48.85	<24.4	<12.2	<12.2
<12.2	<24.4	<3.05	<195.5	<6.1	<3.05	<24.4	<48.85	<24.4	<12.2	<12.2
<12.2	<24.4	<3.05	<195.5	<6.1	<3.05	<24.4	<48.85	<24.4	<12.2	<12.2
<12.2	<24.4	<3.05	<195.5	<6.1	<3.05	<24.4	<48.85	<24.4	<12.2	<12.2
<12.2	<24.4	<3.05	<195.5	<6.1	<3.05	<24.4	<48.85	<24.4	<12.2	<12.2
<12.2	<24.4	<3.05	<195.5	<6.1	<3.05	<24.4	<48.85	<24.4	<12.2	<12.2
<12.2	<24.4	<3.05	<195.5	<6.1	<3.05	<24.4	<48.85	<24.4	<12.2	<12.2
<12.2	<24.4	<3.05	<195.5	<6.1	<3.05	<24.4	<48.85	<24.4	<12.2	<12.2
<12.2	<24.4	<3.05	<195.5	<6.1	<3.05	<24.4	<48.85	<24.4	<12.2	<12.2
<12.2	<24.4	<3.05	<195.5	<6.1	<3.05	<24.4	<48.85	<24.4	<12.2	<12.2
<12.2	<24.4	<3.05	<195.5	<6.1	<3.05	<24.4	<48.85	<24.4	<12.2	<12.2
<12.2	<24.4	<3.05	<195.5	<6.1	<3.05	<24.4	<48.85	<24.4	<12.2	<12.2
<12.2	<24.4	<3.05	<195.5	<6.1	<3.05	<24.4	<48.85	<24.4	<12.2	<12.2
<12.2	<24.4	<3.05	<195.5	<6.1	<3.05	<24.4	<48.85	<24.4	<12.2	<12.2

**Vitexin**	**Secoisolariciresinol**	**Matairesinol**	**Chlorogenic acid**	**Myricetin**	**Isorhamnetin**	**Quercetin**	**Chrysoeriol**	**Epicatechin**	**Catechin**
<3.05	<24.4	<24.4	<3.05	<1562.5	<1562.5	<1562.5	<1.525	<24.4	<24.4
<3.05	<24.4	<24.4	<3.05	<1562.5	<1562.5	<1562.5	<1.525	<24.4	<24.4
<3.05	<24.4	<24.4	<3.05	<1562.5	<1562.5	<1562.5	<1.525	<24.4	<24.4
<3.05	<24.4	<24.4	<3.05	<1562.5	<1562.5	<1562.5	<1.525	<24.4	<24.4
<3.05	<24.4	<24.4	<3.05	<1562.5	<1562.5	<1562.5	<1.525	<24.4	<24.4
<3.05	<24.4	<24.4	<3.05	<1562.5	<1562.5	<1562.5	<1.525	<24.4	<24.4
<3.05	<24.4	<24.4	<3.05	<1562.5	<1562.5	<1562.5	<1.525	<24.4	<24.4
<3.05	<24.4	<24.4	<3.05	<1562.5	<1562.5	<1562.5	<1.525	<24.4	<24.4
<3.05	<24.4	<24.4	<3.05	<1562.5	<1562.5	<1562.5	<1.525	<24.4	<24.4
<3.05	<24.4	<24.4	<3.05	<1562.5	<1562.5	<1562.5	<1.525	<24.4	<24.4
<3.05	<24.4	<24.4	<3.05	<1562.5	<1562.5	<1562.5	<1.525	<24.4	<24.4
<3.05	<24.4	<24.4	<3.05	<1562.5	<1562.5	<1562.5	<1.525	<24.4	<24.4
<3.05	<24.4	<24.4	<3.05	<1562.5	<1562.5	<1562.5	<1.525	<24.4	<24.4
<3.05	<24.4	<24.4	<3.05	<1562.5	<1562.5	<1562.5	<1.525	<24.4	<24.4
<3.05	<24.4	<24.4	<3.05	<1562.5	<1562.5	<1562.5	<1.525	<24.4	<24.4
<3.05	<24.4	<24.4	<3.05	<1562.5	<1562.5	<1562.5	<1.525	<24.4	<24.4

Rutin	Apiin	Amentoflavone	Quer-3-O-Glc	Hyperoside	Epigallo catechin gallate	Kaem-3-O-Glc	Quercitrin	Lut-7-O-Glc	Bajkalin	Api-7-O-Glc
<6.1	<3.05	<3.05		<1.525	<1562.5	<6.1	<3.05	<1.525	<12.2	<3.05
<6.1	<3.05	<3.05		<1.525	<1562.5	<6.1	<3.05	<1.525	<12.2	<3.05
<6.1	<3.05	<3.05		<1.525	<1562.5	<6.1	<3.05	<1.525	<12.2	<3.05
<6.1	<3.05	<3.05		<1.525	<1562.5	<6.1	<3.05	<1.525	<12.2	<3.05
<6.1	<3.05	<3.05		<1.525	<1562.5	<6.1	<3.05	<1.525	<12.2	<3.05
<6.1	<3.05	<3.05		<1.525	<1562.5	<6.1	<3.05	<1.525	<12.2	<3.05
<6.1	<3.05	<3.05		<1.525	<1562.5	<6.1	<3.05	<1.525	<12.2	<3.05
<6.1	<3.05	<3.05		<1.525	<1562.5	<6.1	<3.05	<1.525	<12.2	<3.05
<6.1	<3.05	<3.05		<1.525	<1562.5	<6.1	<3.05	<1.525	<12.2	<3.05
<6.1	<3.05	<3.05		<1.525	<1562.5	<6.1	<3.05	<1.525	<12.2	<3.05
<6.1	<3.05	<3.05		<1.525	<1562.5	<6.1	<3.05	<1.525	<12.2	<3.05
<6.1	<3.05	<3.05		<1.525	<1562.5	<6.1	<3.05	<1.525	<12.2	<3.05
<6.1	<3.05	<3.05		<1.525	<1562.5	<6.1	<3.05	<1.525	<12.2	<3.05
<6.1	<3.05	<3.05		<1.525	<1562.5	<6.1	<3.05	<1.525	<12.2	<3.05
<6.1	<3.05	<3.05		<1.525	<1562.5	<6.1	<3.05	<1.525	<12.2	<3.05
<6.1	<3.05	<3.05		<1.525	<1562.5	<6.1	<3.05	<1.525	<12.2	<3.05

**PSH extracts**
IT F 7
IT M 14
SRB F 7
IT M 7
**EtOH extracts**
IT F 14
IT F 21
IT M 14
IT M 21
IT M 28
SRB F 7
SRB F 14
SRB F 21
SRB F 28
SRB M 7
SRB M 14
SRB M 21
